# The status of the precommissural and postcommissural fornix in normal ageing and mild cognitive impairment: An MRI tractography study

**DOI:** 10.1016/j.neuroimage.2015.12.055

**Published:** 2016-04-15

**Authors:** Kat Christiansen, John P. Aggleton, Greg D. Parker, Michael J. O'Sullivan, Seralynne D. Vann, Claudia Metzler-Baddeley

**Affiliations:** a Cardiff University Brain Research Imaging Centre (CUBRIC), School of Psychology, Cardiff University, Tower Building, 70, Park Place, Cardiff CF10 3AT, UK; b Department of Basic and Clinical Neurosciences, Institute of Psychiatry, Psychology & Neuroscience, King's College London, UK

**Keywords:** Aging, Mild cognitive impairment, Tractography, Fornix, Memory

## Abstract

The fornix connects the hippocampal formation with structures beyond the temporal lobe. Previous tractography studies have typically reconstructed the fornix as one unified bundle. However, the fornix contains two rostral divisions: the precommissural fornix and the postcommissural fornix. Each division has distinct anatomical connections and, hence, potentially distinct functions. Diffusion weighted MRI and spherical deconvolution based tractography were employed to reconstruct these separate fornix divisions and to examine their microstructural properties in both healthy ageing and Mild Cognitive Impairment (MCI). Reliable reconstructions of precommissural and postcommissural fibres were achieved in both groups, with their fibres retaining largely separate locations within the anterior body of the fornix. Ageing and MCI had comparable effects on the two segments. Ageing was associated with changes in mean, axial and radial diffusivity but not with alterations of fibre population-specific diffusion properties, estimated with the hindrance modulated orientational anisotropy (HMOA). Individual HMOA variation in postcommissural, but not precommissural, fibres correlated positively (and unrelated to age) with visual recall performance. This provides novel evidence for a role of postcommissural fibres, which connect structures of the extended hippocampal network, in episodic memory function. Separating the fornix into its two principal divisions brings new opportunities for distinguishing different hippocampal networks.

## Highlights

• Fornix has two rostral divisions with distinct anatomical connections
• New tractography protocol for separating precommissural and postcommissural fornix
• Positive correlation between postcommissural HMOA and episodic memory
• Age-related increases in diffusivity but not in HMOA of fornix
• Mild Cognitive Impairment affects microstructure in both divisions

## Introduction

The fornix is the principal tract linking the hippocampal formation with sites beyond the temporal lobe. Numerous clinical studies have shown how fornix damage disrupts episodic memory, confirming the importance of these hippocampal connections for cognition (Gaffan and Gaffan, 1991, Hodges and Carpenter, 1991, D'Esposito et al., 1995, McMackin et al., 1995, Aggleton et al., 2000, Tsivilis et al., 2008, Vann et al., 2008). This same association is reinforced by MRI-based tractography studies of young (Rudebeck et al., 2009) and older (Metzler-Baddeley et al., 2011) healthy participants. In these MRI studies, indices of fornix microstructure were correlated with episodic memory performance (Rudebeck et al., 2009, Metzler-Baddeley et al., 2011). It is unsurprising, therefore, that amnestic Mild Cognitive Impairment (MCI), which disproportionately affects episodic memory (Albert et al., 2011), should consistently compromise the fornix according to diffusion tensor MRI tractography (Metzler-Baddeley et al., 2012a, Oishi et al., 2012, Zhuang et al., 2012).

**Fig. 1:**
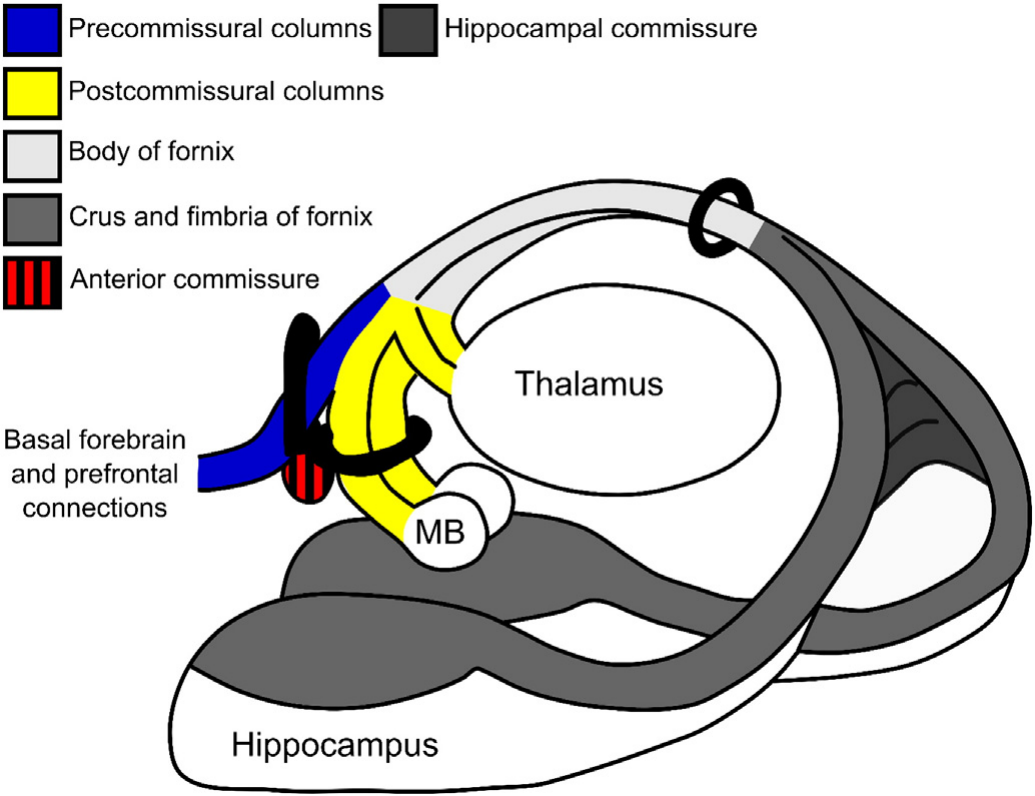
Schematic of the principal components of the fornix and the main areas to which it connects. The black rings represent the placement of the various AND and NOT gates used for the tract reconstructions, as well as the seed ring around the body of the fornix. Abbreviations: MB, mammillary bodies.

The majority of previous studies into age and disease related changes in fornix microstructure, including their relationships with episodic memory, have treated the fornix as a unified tract. Anatomical studies show, however, that the rostral fornix separates into two main branches, split by the anterior commissure (Poletti and Creswell, 1977; Fig. 1). The precommissural fornix principally innervates the basal forebrain (including the septum), ventral striatum and prefrontal cortex, as well as containing fibres projecting from the septum to the hippocampus. Meanwhile, the postcommissural fornix principally innervates the anterior thalamus and the mammillary bodies (Poletti and Creswell, 1977, Aggleton, 2012) and, hence, provides crucial connections between structures of an extended hippocampal network thought to be critical for episodic memory (Aggleton and Brown, 1999).

In response to the evidence for this tract division, the first aim of the present research was to develop an anatomically guided protocol for the *in vivo* reconstruction of precommissural and postcommissural fornix fibres in humans. Separating the reconstructions of precommissural and postcommissural fornix fibres should provide a useful tool for investigating any functional dissociations between these different hippocampal networks, as well as for studying age and disease related effects on these distinct systems. By reconstructing precommissural and postcommissural fibres, it also becomes possible to examine whether these different fibre populations intermingle within the body of the fornix or whether they retain distinct topographies.

Two previous diffusion tensor imaging (DTI) studies have distinguished the precommissural from the postcommissural fornix (Yeo et al., 2013, Chen et al., 2015). The first study used probabilistic tractography to reconstruct the two segments in a group of young, healthy participants (Yeo et al., 2013). The authors reported larger levels of fractional anisotropy (FA), an index of white matter coherence and diffusion directionality, and reduced levels of mean diffusivity (MD) (average diffusion in all directions) in the postcommissural compared to the precommissural fornix (Yeo et al., 2013). The second study divided the fornix into six subregions of interest (precommissural and postcommisural fornices, column, body, crus and fimbria) and found age-related changes in radial diffusivity (RD), i.e., the diffusion perpendicular to the fibres, and axial diffusivity (AD), i.e., the diffusion along the fibres, in all subregions (Chen et al., 2015).

The present study extended these previous findings by investigating the effects of both ageing and neurodegeneration on precommissural and postcommissural white matter microstructure in the fornix of healthy older adults (53–93 years of age) and a group of individuals with MCI. We employed deterministic tractography based on the modified damped Richardson–Lucy (dRL) algorithm for spherical deconvolution that allows, in contrast to conventional DTI, tracking through areas of complex fibre architecture and regions affected by isotropic partial volume (Dell'Acqua et al., 2010). Cerebrospinal fluid (CSF) based partial volume artefacts are of concern for the fornix since this tract is surrounded by the lateral and third ventricles **and volume artefacts may be accentuated** by age- and disease-related atrophy (Metzler-Baddeley et al., 2012b). Based on dRL it was also possible to calculate the hindrance modulated orientational anisotropy (HMOA), a novel index of white matter microstructural organization, defined as the absolute amplitude of the fibre orientation distribution (Dell'Acqua et al., 2013). The HMOA provides a fibre population-specific index of the diffusion properties along the reconstructed fibres that has been shown to be sensitive to individual variation in white matter microstructural organization (Chechlacz et al., 2015) and has been proposed to be more sensitive to changes in diffusion than conventional DTI metrics (Dell'Acqua et al., 2013).

In the present study we excluded fibres of the crus and the fimbria of the fornix from our precommissural and postcommissural fornix reconstructions. The intention was to minimise partial volume effects between the two fibre populations given the fibre crossing and intermingling that must occur between the columns of the fornix and the crus and fimbria of the fornix (Saunders and Aggleton, 2007).

To isolate the two divisions of the fornix, it was necessary to seed different groups of fibres as they descend close to the anterior commissure in the columns of the fornix. At this level, the precommissural and postcommissural divisions contain roughly similar numbers of fibres (Daitz, 1953, Powell et al., 1957). It can be anticipated that the postcommissural reconstructions predominantly involved the connections of the hippocampus with the hypothalamus, including the mammillary bodies (Poletti and Creswell, 1977, Aggleton et al., 2005; Fig. 1). Although the postcommissural fornix also contains many hippocampal projections to the anterior thalamic nuclei (Aggleton et al,. 1986), these fibres were largely excluded from the present study as they turn caudally into the rostral thalamus, just as the columns of the fornix begin to descend (Poletti and Creswell, 1977; Fig. 1).

White matter microstructure in the precommissural and postcommissural fornix segments was investigated with the tract-specific average HMOA index and DTI-based indices of FA, MD, RD and AD (Pierpaoli and Basser, 1996). All DTI-based indices were corrected for CSF-based, partial volume artefacts with the Free Water Elimination (FWE) method (Pasternak et al., 2009). The FWE method also generates a measure of tissue volume fraction (*f*), an index that reflects the volume remaining in each voxel attributable to tissue after the elimination of free water (Pasternak et al., 2009, Metzler-Baddeley et al., 2012b).

The first aim of our study was to demonstrate that it was possible to separate the precommissural fornix from the postcommissural fornix. Secondly, we investigated correlations between the microstructural indices for the two fornix segmentations in a group of healthy older adults, allowing us to investigate age-related effects on microstructure. Thirdly, we studied potential MCI related effects on white matter microstructure in the two segments. Fourthly, we studied whether individual differences in the microstructure of the postcommissural and precommissural fornix fibres were related to differences in episodic memory performance.

## Methods

### Participants

The MRI and cognitive data comprised part of a project into healthy and pathological ageing (MCI) (see Metzler-Baddeley et al., 2011, Metzler-Baddeley et al., 2012a).

#### Healthy ageing cohort

A total of 44 control participants were recruited through advertisements in the local community, GP waiting rooms, newsletters, mail, and via the School of Psychology Community Panel at Cardiff University (Metzler-Baddeley et al., 2011). Participants were between 53 and 93 years of age (mean age 67.7, standard deviation 8.6) and 22 were females. Exclusion criteria were: a history of neurological disease or mental disorder [Clinical Disorders or Acute Medical Conditions/Physical Disorders in the Diagnostic and Statistical Manual of Mental Disorders (DSM-IV-TR)], including a past history of moderate to severe head injury, prior or current alcohol and/or drug abuse, symptomatic memory or other cognitive function decline, previous stroke or cerebral hemorrhage, significant vascular disease elsewhere (peripheral vascular disease, carotid or vertebral artery stenosis or previous coronary intervention), structural heart disease or heart failure, and contra-indications to MRI. Five participants were later excluded due to ill health, motion artefacts, white matter hyper-intensities, or incomplete scan data. These exclusions left 39 control participants in the present study.

#### MCI group and their matched controls

Patients in the MCI group were recruited through the Cardiff Memory Clinic. All patients underwent a standard memory clinic assessment including clinical history, ascertainment of vascular risk factors, full neurological examination, basic hematology and biochemistry investigations, neuroimaging with CT or MRI and cognitive screening with the Addenbrooke's Cognitive Examination (ACE) (Mioshi et al., 2006). Objective memory impairment was confirmed by a score more than 1.5 standard deviations (SD) below age-matched controls on either the ACE verbal memory subscore (Mioshi et al., 2006) or the visual memory test from the Repeatable Battery for the Assessment of Neurological Status (RBANS) (Randolph et al., 1998). All patients had a Mini Mental State Examination (MMSE) (Folstein et al., 1975) score of 24 or above (Mean = 26; SD = 1.7) and a Clinical Dementia Rating of 0.5 (Morris, 1993). The diagnosis of MCI was based on current standard criteria (Albert et al., 2011).

The exclusion criteria for the MCI patients overlapped with those for the healthy ageing cohort (see above). In addition, no patient met diagnostic criteria or had characteristic cognitive or behavioral features to suggest other degenerative disorders such as frontotemporal lobar degeneration, corticobasal degeneration, or dementia with Lewy bodies. All MCI patients demonstrated episodic memory impairments as assessed with the Free and Cued Selective Reminding Test (FCSRT; Grober et al., 1997) and the Doors and People Test (Baddeley et al., 1994). Additional executive dysfunctions were evident in seven patients (see Metzler-Baddeley et al., 2012b). The final 24 participants (11 females) ranged from 58 to 90 years of age with a mean age of 76.7 (SD 7.5).

A subgroup of 20 control participants (10 females) was selected from the healthy aged cohort based on demographic variables and verbal intelligence, in order to provide an age and verbal-IQ Matched Control group for the MCI patients. These Matched Controls were all between 66 and 93 years of age (mean age 74, SD 6.5) and had verbal IQ scores no higher than two standard deviations above the average patient in the National Adult Reading Test (NART; Nelson and Willison, 1991).

### Diffusion-weighted MRI and T_1_-weighted MRI scanning protocols

The diffusion-weighted MRI data were acquired at the Cardiff University Brain Research and Imaging Centre (CUBRIC) with a 3T GE HDx MRI system (General Electric Healthcare). A twice-refocused spin-echo echo-planar imaging sequence provided whole oblique axial (parallel to the inter-commissural plane) brain coverage. Data acquisition was peripherally gated to the cardiac cycle. These data comprised 60 slices of 2.4 mm thickness, with a field of view of 23 cm and a 96 × 96 acquisition matrix, with an echo delay time (TE) of 87 ms and parallel imaging with an ASSET factor of two. The b-value was 1200 s/mm^2^. Diffusion information was encoded along 30 isotropically distributed directions with three additional non-diffusion-weighted scans according to an optimized gradient vector scheme (Jones et al., 1999). The total acquisition time was ~ 13 min per participant. Further, anatomical data were acquired with a T_1_-weighted 3D Fast Spoiled Gradient echo (FSPGR) scan using an oblique-axial acquisition plane with 1 mm isotropic resolution. The matrix size was 256 × 192 × 176 mm (zero-padded to 256 × 256 × 176 mm). In addition, the TR/TE was 7.9/3.0 ms, the TI was 450 ms, and the flip angle was 20^0^. The acquisition time was ~ 7 min.

The diffusion-weighted data were corrected for distortions induced by the diffusion-weighted gradients, artefacts due to head motion, and EPI-induced geometrical distortions by registering each image volume to their T_1_ –weighted anatomical images, which were down-sampled to a resolution of 1.5 × 1.5 × 1.5 mm (Irfanoglu et al., 2012, Pierpaoli, 2011), with appropriate reorientation of the encoding vectors (Leemans and Jones, 2009) in ExploreDTI (Version 4.8.3) (Leemans et al., 2009). A two compartment model using the FWE approach (Pasternak et al., 2009) was then fitted to derive maps of FA, MD, RD and AD (Pierpaoli & Basser, 1996), together with a map of the tissue volume fraction *f* in each voxel (Metzler-Baddeley et al., 2012a).

### Tractography and tract-specific measures

Fornix fibres were reconstructed using ExploreDTI version 4.8.3 (Jeurissen et al., 2011, Leemans et al., 2009) with in-house modifications to support the modified damped Richardson–Lucy (dRL) spherical deconvolution method (Dell'Acqua et al., 2010). Spherical deconvolution based tracking algorithms permit extraction of multiple peaks in the fibre orientation density function (fODF) in voxels with complex fibre architecture and, hence, allow tracking through crossing and kissing fibres. The dRL method additionally improves tracking by reducing isotropic partial volume effects that can degrade spherical deconvolution results. To reconstruct fibre tracts, dRL fODFs were estimated at the center of each image voxel and seed points positioned at the vertices of a 2 × 2 × 2 mm grid superimposed over the image. The tracking then interpolated local fODF estimates at each seed point and propagated 0.5 mm along orientations indicated by up to four supra 0.05 fODF magnitude threshold peaks (ignoring one per axially symmetric pair), allowing four potential streamlines to emanate from each seed point. Individual streamlines were then propagated by interpolating the fODF at their new location and propagating 0.5 mm along the minimally subtending fODF peak, repeating this process until the minimally subtending peak magnitude fell below 0.05 or the change of direction between successive 0.5 mm steps exceeded 45°. This procedure was then repeated by tracking in the opposite direction from the initial seed point. Streamlines outside a minimum of 10 mm and maximum of 500 mm length were discarded. At each 0.5 mm step local estimates of FA, RD, MD, AD and *f* were acquired through interpolation of associated parameter maps while HMOA was captured at the time of streamline generation by recording, with appropriate normalisation (Dell'Acqua et al., 2013), the minimally subtending local fODF peak magnitude.

### Regions of interest (ROI)

Three dimensional fibre reconstructions of the precommissural and postcommissural fornix segments were obtained by applying waypoint region of interest (ROI) gates (“AND”, “OR” and “NOT” gates following Boolean logic) to isolate specific tracts from the whole brain tractography data. ROIs were drawn manually by one operator (KC) who was blind to the group membership of each dataset on direction-encoded colour maps in native space. ROI placement was guided by a series of anatomical landmark protocols.

**Fig. 2:**
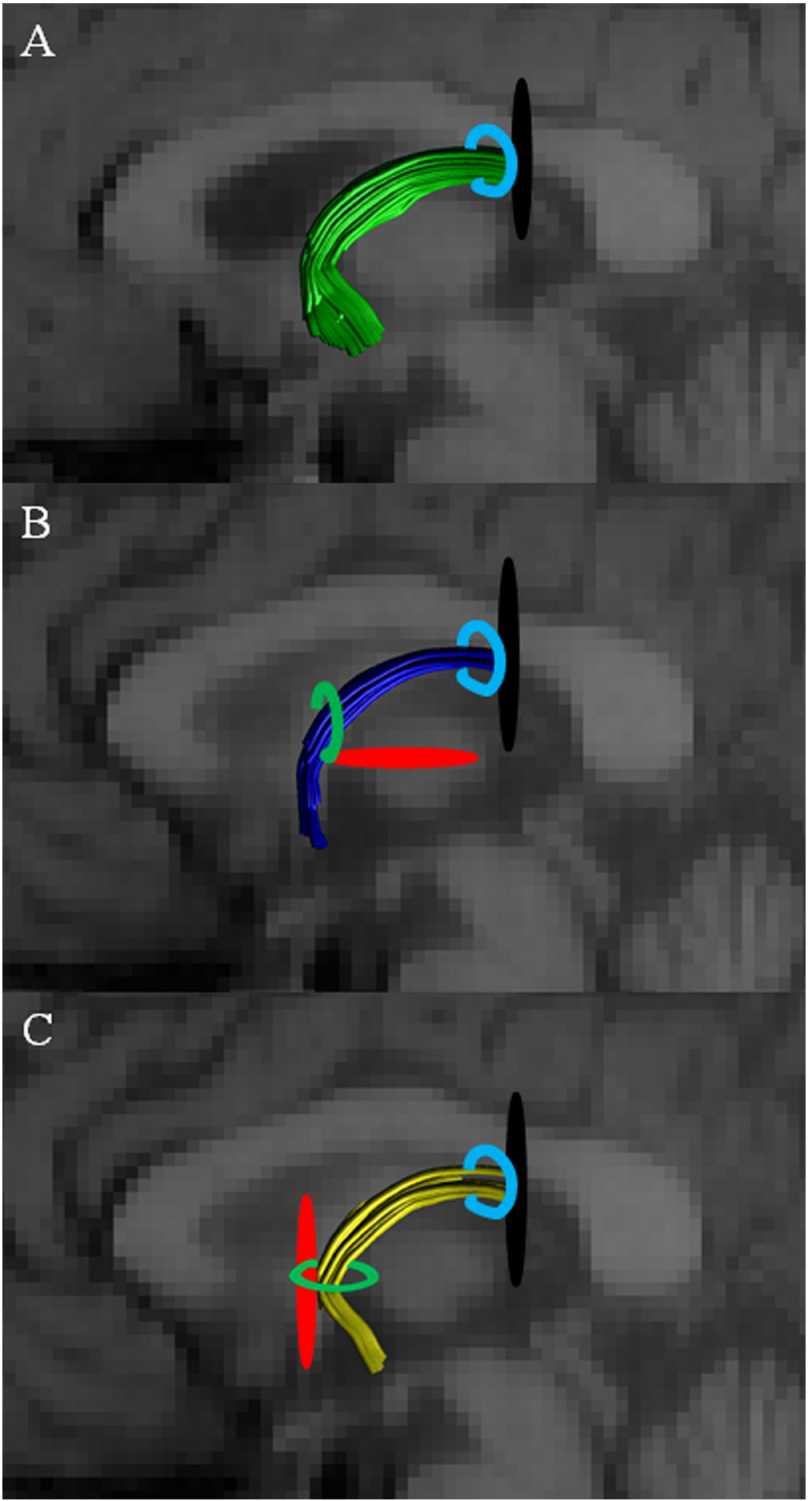
Depictions of the region of interest (ROI) placements on midsagittal slices to obtain: (A) the anterior body of the fornix (abFornix, green), (B) the precommissural fornix and associated fibres (dark blue), and the (C) postcommissural fornix and associated fibres (yellow). Splitter tool placement is indicated in black, NOT gates are in red, SEED gates are in light blue and AND gates are in light green.

The initial task was to reconstruct the anterior body of the fornix (abFornix; Fig. 2A), which included fibres from both the precommissural and postcommissural fornices. This first step involved drawing a seed region encompassing the body of the fornix, six slices posterior to the coronal slice containing the anterior commissure. Fibres not consistent with the known fornix anatomy were excluded from the reconstructions by placing NOT gates on: i. coronal slices immediately anterior to the genu of the corpus callosum and immediately posterior to the splenium of the corpus callosum; ii. axial slices at the level of the lower limit of the body of the corpus callosum and at the level of the upper limit of the pons; and iii. sagittal slices lateral to the fornix at the edge of the medial temporal lobe for each hemisphere.

The precommissural fornix and its component fibres were then distinguished by adding an “AND” ROI just in front of the anterior commissure on a coronal plane, thus selecting the tracts that extend anterior to the anterior commissure (Fig. 2B). A “NOT” ROI was also used, this time on an axial plane, posterior to the anterior commissure, to ensure no overlap occurred between the two tract divisions. The postcommissural fornix reconstructions followed the exact same protocol as outlined above, but with the “AND” and “NOT” ROIs reversed so that only tracts running posterior to the anterior commissure were included (Fig. 2C). The delineation took advantage of how the precommissural and postcommissural fibres separate at the anterior columns of the fornix. To minimise overlap, reconstructions of the precommissural and postcommissural division only included fibres up to the crus of the fornix (Fig. 1), i.e., fibres bending downwards towards the medial temporal lobes and hippocampus were excluded using tract segmentation tools (the “splitter” tool) within ExploreDTI 4.8.3. This procedure was also chosen to avoid tract “jumping” in areas where tracts pass close or across each other, whereby one tract voxel “jumps” onto a neighboring tract voxel due to the angle/FA threshold being very similar to the neighboring voxel (Jones and Cercignani, 2010).

### Episodic memory

Verbal and visual recall abilities were assessed as part of an ongoing study into ageing and MCI (Metzler-Baddeley et al., 2011, Metzler-Baddeley et al., 2012a), which involved the Free and Cued Selective Reminding Test (FCSRT) (Grober et al., 1997) and the Doors and People Test (Baddeley et al., 1994). Performance on the FCSRT verbal free and total recall (cued and free recall), as well as visual recall in the Doors and People Test correlated most strongly with fornix status in healthy ageing (see Metzler-Baddeley et al., 2011, Table 3). Therefore, the present study focused on the same episodic memory measures.

### Statistical analysis

All statistical analyses employed SPSS v. 20 (IBM Corp, 2011). All microstructural data for each tract were inspected for outliers defined as values larger than three times the absolute z-score from the mean. Based on this criterion, one value for abFornix, precommissural and postcommissural fornix MD, two values for abFornix, precommissural and postcommissural fornix AD and one value for abFornix FA in the MCI group, and two values for postcommissural fornix *f* (one in the MCI cohort and one in their control cohort) were excluded from the data analyses.

#### Assessment of overlap between the precommissural and postcommissural fornix tracts

The overlap between the precommissural and postcommissural fornices was assessed by means of DICE scores computed for each individual (Dice, 1945). The DICE coefficient is essentially a measure of similarity between two samples and was calculated with the following formula (where *x* is the DICE score, *A* and *B* are two separate samples and *C* is the overlap between them): *x* = 2*C*/(*A* + *B*). DICE coefficients vary between 0 and 1, the higher the coefficient the higher the level of overlap between the samples.

To provide additional visualisation of the overlap between the two segments, participants' T_1_-weighted images were non-linearly co-registered to the Montreal Neurological Institute (MNI) 1x1x1mm template allowing, through reuse of the warp fields, the transformation of individual participant's precommissural and postcommissural fornix tract masks (3D volumes in which voxels intersected by a streamline are set to 1, all others zero) into a common space. Individual voxels were then assigned a probability of belonging to the precommissural and postcommissural fornix segments. To delineate a boundary between the two structures a simple voting scheme was employed in which voxels were assigned to the fornix segment with the highest probability based on the proportion of participants whose streamlines had passed through that (spatially normalised) location.

#### Assessment of white matter microstructure in the precommissural and postcommissural fornix tracts: effects of age and MCI

Pearson's r correlation coefficients were calculated between the white matter microstructural indices (HMOA, FA, RD, MD, AD, and *f*) derived for the abFornix, precommissural fornix, and postcommissural fornix to determine their degree of co-linearity. For the Healthy ageing cohort (n = 39) correlations were also calculated with age. It is appreciated that the measures derived from the abFornix fornix are not independent from those of the precommissural or postcommissural fornix, indeed the abFornix is largely comprised of these two components. Nevertheless, these correlations indicate the extent to which the two divisions of the fornix provide proxy measures for more complete tract reconstructions. Bonferroni correction was applied to the 36 correlations to account for multiple comparison error, leading to family-wise alpha level of 0.0014 for each separate correlational analysis.

To investigate whether white matter microstructure in abFornix, as well as in the precommissural and postcommissural fornices, was differentially affected by MCI, we first conducted independent t-tests between the two groups for HMOA, FA, RD, MD, AD, and *f* in the abFornix separately. The Bonferroni corrected alpha level for these six comparisons was ≤ 0.008.

Subsequent analyses of variance (ANOVA) involved the between-factor of group (controls and MCI) and the within-factor of tract (precommissural and postcommissural fornix) for each microstructural measure (HMOA, FA, RD, MD, AD, and *f*) separately. (The abFornix was not included in these ANOVAs as it contains both precommissural and postcommissural fibres and, hence, is not independent.) Significant interactions (p ≤ 0.05) were followed up with simple effects tests, which used the pooled error term (Winer, 1971).

To assess correlations between white matter microstructure and episodic memory, Pearson's correlation coefficients r were computed between the microstructural indices (HMOA, FA, RD, MD, AD, *f*) for each tract division and the three episodic memory measures (FCSRT verbal free recall, FCSRT total recall Doors and People immediate visual recall). The effects of ageing on these correlations were also tested by partialling out age for both the healthy ageing and MCI cohorts. For each group there were 36 correlations in total (between six microstructural indices for the two segments and the three memory scores). Bonferroni correction was applied for each group separately and only findings surviving the multiple comparison adjustment (alpha level of 0.0014) are reported, unless otherwise stated. Greenhouse–Geisser corrected values are reported when the data violate the assumption of sphericity.

## Results

**Fig. 3:**
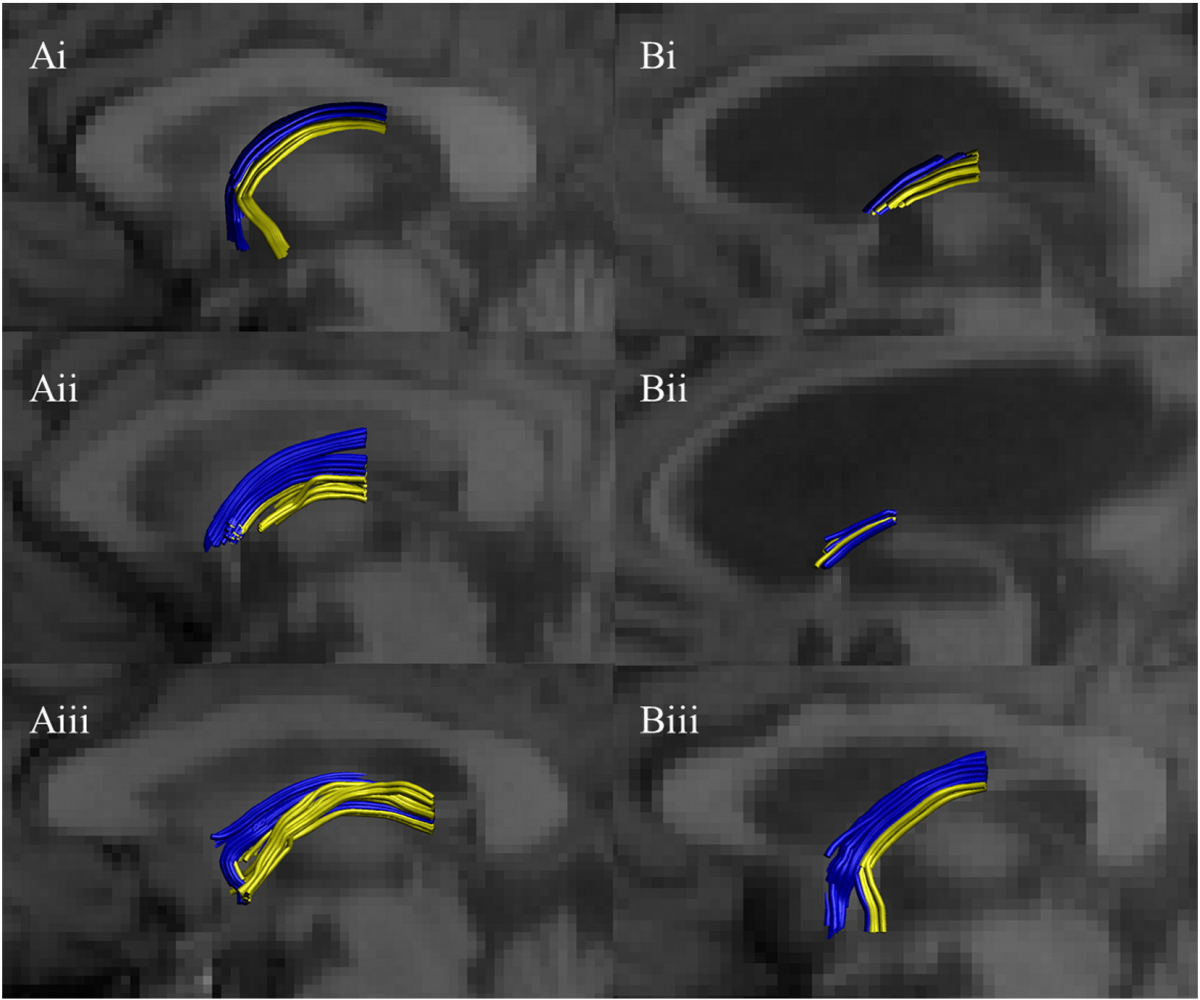
Three examples of tract reconstructions on midsagittal slices from: (A) the Healthy ageing group, and (B) the Mild Cognitive Impairment group. The precommissural fornix is blue and the postcommissural fornix is yellow.

The precommissural and postcommissural fornices could be reconstructed for all participants in both the Healthy ageing cohort and the MCI patients (Fig. 3). However, visual inspection of the reconstructions revealed much variability in tract trajectories in both groups. While many reconstructions involved the columns of the fornix, with clear evidence of an anterior–posterior split, some reconstructions stopped at the beginning of the columns or were less clearly divided by the anterior commissure (Fig. 3). Evidence that MCI affects these tracts came from group comparisons of the length of the white matter reconstructions. For all three tract reconstructions, the mean length of fibres was lower for patients than their matched controls (abFornix, t(42) = 2.20, p = 0.033; precommissural fornix, t(42) = 3.37, p = 0.002; postcommissural fornix, t(42) = 3.85, p = 0.0003).

### Overlap of precommissural and postcommissural fornix: healthy ageing cohort (n = 39)

The mean DICE coefficient between the precommissural and postcommissural fornix was 0.28 (SD 0.19), with a range from 0 to 0.64 (where a score of 1 would reflect complete overlap). The median coefficient score was 0.24.

**Fig. 4:**
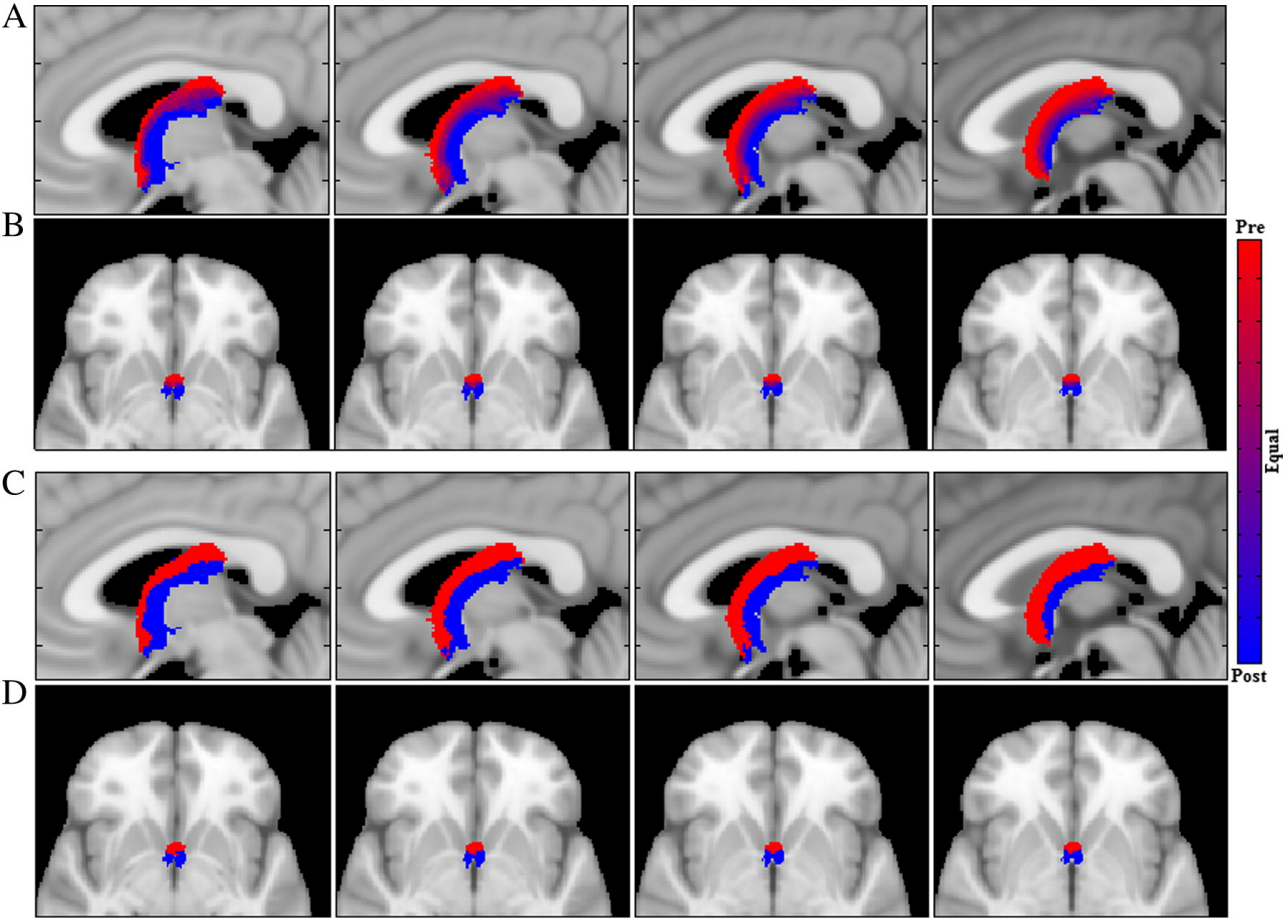
(A–B) Relative probability maps identifying voxels as belonging to the precommissural (red) and postcommissural (blue) fornix bundles with image sequence (A) traversing from left lateral to the midline plane (MNI 1 mm y-axis slices 87–90) and (B) traversing superiorly from MNI z-axis slice 66 to 70. (C–D) Binary segmentations of A and B according to the voting scheme described in the Statistical analysis section.

A visual representation of the overlap of the precommissural and postcommissural tracts can be seen in Fig. 4. Specifically, Fig. 4a and b show the relative probability of voxels belonging to either tract while Fig. 4c and d binarize those maps according to the voting scheme described in the Assessment of overlap between the precommissural and postcommissural fornix tracts section. Note that while overlap does exist (as displayed by the purple hues in Fig. 4a and b) the two structures remain clearly separable (this is particularly apparent in the transverse plane) and demonstrate good agreement with the known anatomy.

### Correlations between precommissural and postcommissural fornix diffusion MRI measures: healthy ageing cohort (n = 39)

In the Healthy ageing cohort, all of the abFornix indices correlated significantly with their precommissural and postcommissural counterparts. In contrast, for those correlations between the precommissural and postcommissural divisions, only the RD, MD and AD values correlated significantly (Table 1). Furthermore, age correlated with RD, MD and AD, but not with HMOA, FA or *f*, for the abFornix and postcommissural fornix. For the precommissural fornix, trends (significant at uncorrected level) for correlations between age and RD, MD and AD were observed (Table 2).

### Correlations between the anterior body of the fornix (abFornix), precommissural fornix, and postcommissural fornix diffusion MRI measures: mild cognitive impairment (n = 24) and matched controls (n = 20)

In the MCI group, there were significant correlations between FA, MD, RD and *f* in the abFornix and the precommissural fornix but no correlations were observed for AD and HMOA (Table 3). All but the HMOA and *f* measures correlated between the postcommissural and abFornix for the MCI patients (Table 3). Correlations between the precommissural and postcommissural fornices of the MCI group were significant for FA, RA and MD.

In the Matched Control group, all of the abFornix diffusion measures correlated with their corresponding postcommissural fornix diffusion measures (Table 3). For the corresponding comparisons between the abFornix and precommissural fornix, RD, MD and AD were significantly correlated (Table 3). Again, between the precommissural and postcommissural fornix, RD, MD and AD were all significantly correlated but no significant correlations were observed for HMOA, FA and *f* (Table 3).

### Comparisons between mild cognitive impairment (n = 24) matched controls (n = 20) for tract measures

There were trends (significant at the uncorrected level) for larger FA [t(41) = 2.43, p = 0.02] and larger HMOA [t(42) = 2.66, p = 0.01] in the abFornix for the Matched Controls compared to the MCI patients (Table 4). No significant group effects were observed for RD, MD, AD or *f* in the abFornix [RD, t(42) = 0.97, p = 0.83; MD, t(42) = 1.20, p = 0.24; AD, t(42) = 1.35, p = 0.18; and *f*, t(42) = 1.30, p = 0.10].

For the two tract divisions, there was a significant group by tract interaction for FA [F(1, 42) = 6.23, p < 0.05]. Inspection of the data (Table 4), suggests a relative decrease in FA scores for the precommissural fornix of the MCI cases, with a relative increase for the postcommissural fornix. However, follow up simple effects tests were not significant. For RD, there was no interaction [(F(1, 42) = 1.48, p = 0.23] and also no group effect [F(1, 42) = 0.05, p = 0.83]. Neither MD nor AD possessed significant tract by group interactions or main effects of group [F(1, 41) = 0.03, p = 0.87 and F(1, 41) = 0.26, p = 0.62 for MD; and F(1, 40) = 1.12, p = 0.30, and F(1, 40) = 2.03, p = 0.32 for AD, respectively]. While there was also no interaction for *f* (F < 1), there was a main effect of group [F(1, 40) = 8.70, p < 0.01], reflecting lower *f* scores in the MCI patients. No group [F(1, 42) = 0.13, p = 0.11], tract [F(1, 42) = 0.0, p = 0.99] or interaction effects [F(1, 42) = 2.6, p = 0.11] were observed for HMOA.

### Correlations between abFornix, precommissural fornix, and postcommissural fornix diffusion MRI indices and episodic memory measures: healthy ageing cohort (n = 39), mild cognitive impairment (n = 24) and matched controls (n = 20)

**Fig. 5:**
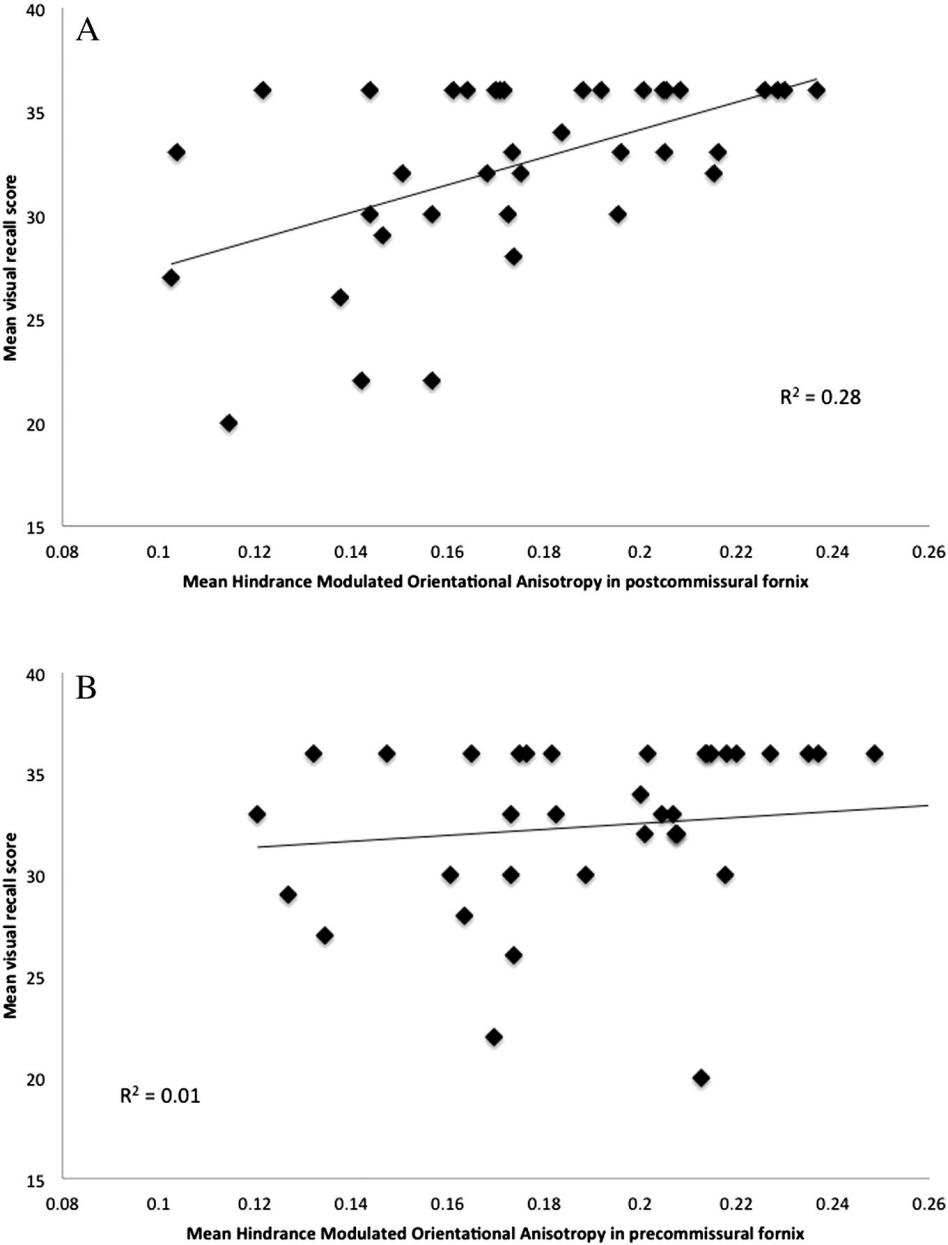
A**.** Positive correlation between performance in the visual recall task of the Doors and People Test and variation in the mean Hindrance Modulated Orientational Anisotropy (HMOA) index in the postcommissural fornix for healthy older adults (n = 39). This correlation remained significant after partialling out age. B. No significant correlation was observed between visual recall performance and HMOA in the precommissural fornix and the two correlations coefficients differed significant from each other z = 1.96 (p = 0.025, 1-tailed).

For the Healthy ageing cohort (n = 39) there was a positive correlation between individual differences in visual recall performance and in HMOA in the postcommissural fornix (r = 0.53, p = 0.001) (see Fig. 5), which remained significant after the partialling out of age (r = 0.5, p = 0.002). No correlation with visual recall was observed for HMOA in the precommissural fornix (r = 0.12, p = 0.47) and a trend (significant at the uncorrected level) was present for HMOA in abFornix (r = 0.34, p = 0.04). Fisher's r to z test demonstrated that the correlation coefficients for the precommissural and postcommissural fornix differed significantly z = 1.96 (p = 0.025, 1-tailed).

A number of trends reflecting negative correlations were observed. These trends included the relationship between abFornix RD and MD and visual recall performance (r = − 0.463, p = 0.003 and r = −.0456, p = 0.004 respectively) and verbal free recall performance (r = − 0.443, p = 0.005 and r = − 0.441, p = 0.005 respectively) and between postcommissural fornix RD and MD and verbal free recall performance (r = −.456, p = 0.004 and r = − 0.441, p = 0.005). However, none of these correlations remained significant after the partialling out of age.

At the same corrected alpha level (p ≤ 0.0014), there were no significant correlations between episodic memory performance and any of the DTI-based indices in each of the three reconstructed tracts for either the MCI or the Matched Control group. However, the Matched Control group showed a trend for a positive correlation between inter-individual differences in visual recall performance and in HMOA in the postcommissural fornix (r = 0.65, p = 0.003), which remained after controlling for age (r = 0.59, p = 0.009). No significant correlation with HMOA was observed for the MCI patients.

## Discussion

The primary aim of the present study was the development and evaluation of an anatomical protocol for the reliable reconstructions of two major subdivisions of the fornix, the precommissural and postcommissural fornix. In contrast to previous studies (Yeo et al., 2013, Chen et al., 2015) we employed deterministic tractography based on the dRL spherical deconvolution algorithm and a fibre-population specific index of diffusion (HMOA), whilst focusing our reconstructions on the columns and the body of the fornix to reduce partial volume between the two segments (Yeo et al., 2013). We successfully demonstrated that it was possible to reconstruct reliably the precommissural and postcommissural segments of the fornix, not only in healthy older adults but also in patients with MCI, a condition known to exhibit significant degeneration in limbic pathways. A secondary aim was to investigate the effects of ageing and neurodegeneration on the microstructure of these two fornical divisions, as well as investigating potential dissociations between microstructural variations in the precommissural versus postcommissural fornix and episodic memory function. Whilst we did not find clear-cut dissociative effects of ageing or MCI on the microstructure of the precommissural and postcommissural fornix, we provide evidence for a specific correlation between white matter microstructure in the postcommissural fornix and visual recall performance that was not observed for the precommissural fornix. The latter result is consistent with the view that postcommissural fornix fibres facilitate communication within the extended hippocampal network and hence play a crucial role in mediating episodic memory function.

### Tractography of precommissural and postcommissural fornix subdivisions

The fornix is of considerable interest as it provides the principal route for projections from the hippocampal formation to the prefrontal cortex, ventral striatum, basal forebrain, hypothalamus, and thalamus, along with return inputs to the hippocampus from the hypothalamus, basal forebrain, and midbrain (Poletti and Creswell, 1977, Swanson et al., 1987, Saunders and Aggleton, 2007). These various hippocampal connections differentially involve the precommissural fornix and postcommissural fornix (Fig. 1). The additional appreciation that the hippocampal formation has multiple functions highlights how these various connections might play different roles (O'Mara, 2005, Fanselow and Dong, 2010, Aggleton, 2012, Strange et al., 2014). For this reason, it would be valuable to distinguish hippocampal connections using *in vivo* imaging methods. This rationale is reinforced by tracer studies in animals showing that individual hippocampal neurons rarely contribute axons to both the precommissural and postcommissural fornix (Donovan and Wyss, 1983, Naber and Witter, 1998).

The first step was to separate the precommissural fornix from the postcommissural fornix. This goal was achieved in all cases, even in those with presumed degenerative pathology (MCI). The two fornix subdivisions were then compared with each other and with a more complete reconstruction of the anterior body of the fornix, the ‘abFornix’. As noted, the abFornix was not simply the sum of the precommissural and postcommissural reconstructions as it could additionally include the hippocampal projections to the anterior thalamus, which were excluded from the postcommissural fornix reconstructions (Fig. 1).

As can be seen from the relative and binarized probability maps depicted in Fig. 4, the present reconstructions show a clear separation between the precommissural and postcommissural fibres, not only in the columns but also in the anterior body of the fornix. We employed a simple voting scheme that assigned each voxel to the segment with the higher probability (relative to the other segment) based on the proportion of participants whose streamlines had passed through that particular location. Whilst there was some overlap (as depicted in purple in Fig. 4a and b) the two segments were clearly separable and demonstrated good agreement with the known anatomy. This tract separation was further supported by the low DICE score between the two fornix divisions.

Despite clear evidence for a separation of fibres within the body of fornix (fibres for the precommissural fornix in dorsal fornix, fibres for the postcommissural fornix in the ventral fornix, see Fig. 4), little has been previously reported about the topography of fibres within the primate fornix. One study of macaque monkeys found that fibres from the more anterior hippocampus preferentially occupy the lateral fornix while posterior hippocampal fibres preferential occupy the medial fornix (Saunders and Aggleton, 2007). As neurons from the entire anterior–posterior length of the hippocampus contribute to the precommissural and postcommissural fornices (Barbas and Blatt, 1995, Carmichael and Price, 1995, Friedman et al., 2002, Aggleton et al., 2005, Aggleton, 2012), there is the clear implication that hippocampal projections realign along the body of the fornix in the dorsal–ventral plane, prior to reaching the columns of the fornix. This same topographic separation may equally occur in those projections to the hippocampal formation via the fornix. For the precommissural fornix, such projections principally arise from the septum (Swanson et al., 1987, Saunders and Aggleton, 2007). Consistent with the above prediction, it has been observed that the septal (precommissural) connections of the primate hippocampus concentrate in the medial fornix, immediately under the callosum (Poletti and Creswell, 1977), i.e., in a dorsal location, consistent with this topography.

### Microstructural properties of precommissural and postcommissural fornix subdivisions

In addition to mapping the various pathways, their microstructure was investigated using DTI-based indices of FA, MD, RD, AD and tissue volume fraction *f* as well as HMOA, a novel fibre population-specific index of white matter organization (Dell'Acqua et al., 2013). DTI-based indices provide average measures of tissue properties, which is problematic when the voxel contains more than one fibre population or different tissues (white matter, gray matter, CSF) since these indices are then no longer fibre population or tissue-specific. For the precommissural and postcommisural tracts this issue creates challenges where, for instance, the anterior columns of the fornix cross the anterior commissure. Voxels in these areas may contain fibres from both the fornix and the commissure and, hence, FA and diffusivity indices will not provide fibre population-specific measures. HMOA overcomes this problem by providing an index more specific to single fibre populations and, hence, provides a purer measure of the microstructural properties of the precommissural and postcommissural fornix in voxels with anterior commissure contamination (Dell'Acqua et al., 2013). Furthermore, the HMOA index incorporates microstructural information including fibre orientation and density, radial diffusion hindrance and anisotropy. For this reason, HMOA may provide a richer and more sensitive index to detect fibre population-specific changes in white matter microstructure (Dell'Acqua et al., 2013).

In the healthy ageing group, all six indices for the abFornix correlated with the corresponding index scores from both the precommissural and postcommissural pathways (Table 1). Similar patterns of correlations between abFornix and the two tract subdivisions were observed for the matched control and the MCI groups although it should be noted that some correlations, for instance for HMOA, did not reach multiple comparison corrected levels of significance. This is very likely due to the reduced sample size in the matched control group and perhaps due to a combination of reduced sample size and reduced fornix functionality in the MCI group (Table 3).

The consistent correlations in the healthy ageing group were expected as the abFornix reconstructions included both of its two divisions. Of more interest, therefore, was the finding that only the RD, MD and AD measures from the precommissural and postcommissural pathways correlated with each other whilst no significant correlations were observed for HMOA, FA and *f* (Table 1). Since HMOA provides a fibre population-specific measure of microstructural properties along the reconstructed tract independent of other diffusion directions (Dell'Acqua et al., 2013), the lack of a significant correlation between HMOA in the precommissural and postcommissural fornix may suggest that these segments are characterized by different and unique diffusion orientations. This observation is consistent with the above results from the DICE analyses and probability maps, which demonstrated a clear anatomical separation of these tracts. In addition, the lack of correlation between the HMOA indices of the two tracts may also reflect differences in terms of microstructural properties such as fibre density or volume fraction and/or the anisotropy of each fibre orientation. This interpretation is further supported by the lack of correlations for FA and the tissue volume fraction.

In contrast, we observed significant positive correlations between MD, AD and RD between the two tracts. MD measures the average diffusion in all directions, AD the diffusion along and RD the diffusion perpendicular to the principal diffusion direction. The positive correlations between the diffusivity indices suggest that diffusion properties in directions other than along the reconstructed fibre orientation, which are captured by the diffusivity indices but not by HMOA, were shared between the two subdivisions. Diffusivity indices provide less specific but potentially more sensitive markers of changes in diffusion due to averaging of many possible alterations including changes in axonal myelin, membrane density and diameter, as well as fibre geometry and dispersion (Pierpaoli and Basser, 1996). Furthermore, although we applied free water correction, we cannot rule out that some CSF based partial volume effects remained and were shared by diffusivity indices from both subdivisions (Metzler-Baddeley et al., 2012a, Metzler-Baddeley et al., 2012b). Thus, the pattern of results suggests that the two tracts differ in microstructural properties of fibre orientation, density, and anisotropy but that “non-fibre population-specific” diffusion properties, for instance, due to atrophy based partial volume effects may be shared. There is a clear need for future studies combining diffusion MRI and histology to validate the biological basis of these imaging markers and to investigate potential microstructural differences between the different fornix fibre populations.

We included DTI-based indices in the present study for the purpose of comparability with previous studies. Yeo et al., (2013) separated the precommissural and postcommissural fornix in young, healthy participants using probabilistic tractography, a technique that provides information about how likely two regions of interest are to be connected with each other. In contrast, the present study adopted a deterministic tractography approach guided by prior knowledge of the trajectory and connectivity of the fornix. Nevertheless, comparisons between the FA estimates for healthy ageing (present study) and for younger participants (Yeo et al., 2013) found no statistical difference in the postcommissural fornix FA values. In contrast, the precommissural FA estimates were significantly higher for the present study, presumably reflecting the different methods and different population groups.

### Age-related effects on the microstructure of precommissural and postcommissural fornix subdivisions

Studies using diffusion MRI tractography already indicate that fibres composing the fornix are affected by normal and abnormal ageing. Fornix microstructure has been shown to correlate with age and to change in MCI and Alzheimer's disease (Metzler-Baddeley et al., 2011, Metzler-Baddeley et al., 2012a, Zhuang et al., 2012, Oishi et al., 2012). However, these previous studies investigated the fornix as a unitary structure whilst the present research explored the effects of ageing and MCI on different fibre populations within the fornix (see also Cheng et al., 2015). For healthy older adults we found positive correlations between age and MD, AD, and RD for the postcommissural fornix and abFornix, whilst no correlations were observed for HMOA, FA, and *f*. Although correlations between age and diffusivity indices did not survive Bonferroni corrections, a similar pattern of results was observed for the precommissural fornix. These results suggest that ageing has comparable effects on all fibres within the fornix.

However, these results also demonstrate that age-related changes are not found universally in all microstructural indices. While ageing was associated with microstructural changes that affect the overall magnitude of diffusivity, caused, for example, by vascular insults, inflammation, demyelination, Wallerian degeneration or atrophy, no age-related effects were observed for fibre population-specific microstructural properties measured with the HMOA index. It could be the case that diffusivity indices capture the total of age-related changes in white matter microstructure better than HMOA because they include information of diffusivity alterations in all directions, including potential atrophy related partial volume effects. Evidence from post-mortem studies indicates that age-related atrophy of white matter in the human brain may be caused by a selective loss of myelinated fibers of small diameter (Tang et al., 1997) but it remains unknown to what extent the fornix bundle is affected by these processes. Again, correlative data between HMOA and other diffusion MRI markers and histology in humans and animals would provide further information about age-related effects on different fibre populations.

In contrast to a previous study (Metzler-Baddeley et al., 2011), we did not find any relationships between age and FA or *f* in those portions of the fornix studied here. This discrepancy presumably arose from the differences in tract reconstruction, e.g., the abFornix reconstruction did not include fibres posterior to the crus. Another methodological difference concerns the adoption of EPI-distortion corrections in the present analyses (Pierpaoli, 2011). Previous studies have reported systematic differences between tract reconstructions before and after EPI reconstruction (Andersson et al., 2004, Lee et al., 2004) with fibres appearing more symmetrical and more consistent with known anatomy after the correction. Thus, EPI correction may result in more precise tract reconstructions and, hence, enhanced sensitivity to RD and AD. This in turn may remove some variation in FA, since FA may remain constant when both AD and RD increase and, therefore, may limit the sensitivity of FA. Indeed, a number of studies have observed that diffusivity indices were more sensitive markers of age- and disease-related changes than FA, potentially for this same reason (Acosta-Cabronero et al., 2010). It should also be noted that Chen et al., (2015) observed age-related changes in RD for the body of the fornix but not for the precommissural and postcommissural columns. We would argue that this apparent dissociation likely reflects differential sensitivities between the larger fibre populations in the body relative to the smaller and thinner fornix columns. Since our reconstructions included both columns and body we cannot infer whether the observed correlation between age and RD in the postcommissural fornix was primarily driven by fibres in the body or the columns.

### MCI related changes in microstructure of the precommissural and postcommissural fornix subdivisions

We did not observe a specific disease-related dissociation between precommissural and postcommissural fibres. Although MCI was associated with a trend for lower HMOA and FA indices for the abFornix and a group by tract (precommissural and postcommissural fornix) interaction was observed for FA, no significant differences were present between patients and Matched Controls in the precommissural and postcommissural segmentations. These results suggest that MCI is associated with pathological processes that affect both fibre populations. Indeed there is evidence both for hippocampal/medial temporal lobe lesions as well as basal forebrain impairment in this patient group (e.g. Grothe et al., 2010, Mufson et al., 2012).

The trends for lower HMOA and FA in the abFornix and lower *f* across both precommissural and postcommissural tracts for MCI patients compared to their matched controls were consistent with previous reports of impaired fornix microstructure in MCI and early Alzheimer's disease (Metzler-Baddeley et al., 2012a, Zhuang et al., 2012, Fletcher et al., 2013, Kantarci, 2014). However, although we observed an interaction between group and fornix tract subdivision for FA, suggesting potential differential effects of MCI pathology across the two pathways, there were no significant group differences in microstructure for either the precommissural or the postcommissural fornix alone. This could be an effect of the small segmentations that may have reduced microstructural variability relative to the abFornix or the total fornix reconstructions in previous studies. Further studies are required to find out if there is a potential link between patterns of pathological change across the two fornix pathways with clinical profiles of symptomatology, rate of disease progression, and treatment response. One proposal would be to compare the status of the fornix tract divisions in pure amnestic MCI versus MCI patients with additional executive deficits (multi-domain MCI).

### Correlations between microstructure in the two tracts and episodic memory

Preliminary evidence was found for a functional distinction between the two fornical segments. Episodic memory performance in the healthy older cohort was positively correlated with variation in the HMOA index of the postcommissural fornix but not the precommissural fornix (Fig. 5). In contrast to the trends for negative correlations between visual recall and diffusivity indices (RD and MD) both in the abFornix and postcommissural fornix, this correlation with postcommissural HMOA was unrelated to age.

Reconstructions of postcommissural fibres will in part reflect hippocampal projections to the mammillary bodies**,** which have been repeatedly implicated in episodic memory (Dusoir et al., 1990, Parker and Gaffan, 1997, Van der Werf et al., 2003, Vann and Aggleton, 2004, Tsivilis et al., 2008, Carlesimo et al., 2015). Other postcommissural connections include the supramammillary projections to the hippocampus (Saunders and Aggleton, 2007), which are proposed to set theta frequency (Kirk, 1998). The separate reconstruction of precommissural and postcommissural fornix fibres will allow future research to investigate more closely the memory functions associated with these fibre segments. For instance, it could be the case that postcommissural fornix fibres are vital for encoding episodic memory whilst precommissural fibre connections with the prefrontal cortex and basal forebrain may play a more specific role in strategic aspects of encoding and retrieval (Baxter and Chiba, 1999, Fletcher and Henson, 2001, Simons and Spiers, 2003, Browning et al., 2005, Easton et al., 2012, Ray et al., 2015).

Memory-relevant projections in the postcommissural fornix are likely to follow a particular orientation due to the anatomical location of the structures they innervate. Therefore we may have observed memory correlations with HMOA that includes information about fibre orientation whilst correlations with the diffusivity indices appeared to have been primarily driven by “non-fibre population-specific” age effects. As already mentioned, the diffusivity indices may have been susceptible to partial volume from other fibres, notably the anterior commissure, and to potential residual isotropic contamination, thus making them less sensitive to fibre population-specific microstructural changes. Indeed simulation studies have shown that properties such as axonal diameter can be better detected with HMOA than FA and MD, and that HMOA may be sensitive to small microstructural changes before they become visible in FA (Dell'Acqua et al., 2013). Regarding the lack of correlations with the FA index, it is possible that age-related increases in diffusivity in all directions may have removed the sensitivity of the FA measure, since FA remains constant when both AD and RD increase (Acosta-Cabronero et al., 2010).

It should be noted that we did not observe significant correlations between individual differences in HMOA and memory performance for the MCI and the matched control groups. The matched control group showed a trend for a positive correlation that most likely did not reach significance due to the reduced sample size. Similarly, sample size may have contributed to the lack of a significant effect in the MCI group. However, a previous study with the same group of participants found that the matched control group exhibited a significant positive correlation between verbal free recall and tissue volume fraction for the fornix tract as a whole whilst such a correlation was absent in the MCI group (Metzler-Baddeley et al., 2012a, Metzler-Baddeley et al., 2012b). Instead, recognition memory correlated with individual differences in tissue volume fraction of the parahippocampal cingulum (PHC) in the MCI but not the control group (Metzler-Baddeley et al., 2012a, Metzler-Baddeley et al., 2012b). Furthermore, the extent of this “shift” from the fornix to the PHC was positively correlated with larger volumes of the basal forebrain and better memory performance in the patient group (Ray et al., 2015). Based on this pattern we proposed that in the presence of a breakdown of fornix functionality in MCI, other structures of the extended medial temporal and basal forebrain system may compensate for that loss. Thus, the lack of a significant correlation between HMOA in postcommissural fornix and episodic memory performance was most likely a result of already-reduced fornix functionality in MCI.

### Summary

The present study developed and evaluated a novel anatomical protocol for the reliable reconstructions of the precommissural and postcommissural fornix based on deterministic tractography using the dRL algorithm. It was possible to reliably reconstruct precommissural and postcommissural segments of the fornix in healthy older adults and patients with MCI. Whilst we did not find clear-cut dissociative effects of ageing or MCI on the microstructure of the precommissural and postcommissural fornix, we provide evidence for a specific correlation between a fibre population-specific index of white matter microstructure (HMOA) in the postcommissural fornix and visual recall performance, which was not observed for the precommissural fornix. The latter result is consistent with the view that postcommissural fornix fibres facilitate communication within an extended hippocampal network that plays a crucial role in episodic memory function.

## Figures and Tables

**Table 1:** Pearson r correlation coefficients between all microstructural indices in the three tracts in the Healthy ageing cohort. Asterisks indicate significant correlations following Bonferroni adjustment (p ≤ 0.0014). The term abFornix refers to the anterior part of the body of the fornix. Abbreviations: FA, fractional anisotropy; HA, hindrance modulated orientational anisotropy (HMOA); Precom, precommissural fornix; postcom, postcommissural fornix; RD, radial diffusivity; MD, mean diffusivity; AD, axial diffusivity; f, tissue volume fraction.

Healthy ageing cohort (n = 39)												
	Precommissural fornix						Postcommissural fornix					
abFornix	HA	FA	RD	MD	AD	*f*	HA	FA	RD	MD	AD	*f*
HA	0.68^⁎^						0.67^⁎^					
FA		0.60^⁎^						0.63^⁎^				
RD			0.91^⁎^						0.88^⁎^			
MD				0.91^⁎^						0.86^⁎^		
AD					0.87^⁎^						0.79^⁎^	
*F*						0.74^⁎^						0.71^⁎^

*Postcommissural fornix*												
HA	0.22											
FA		0.24										
RD			0.75^⁎^									
MD				0.71^⁎^								
AD					0.57^⁎^							
*f*						0.35						

**Table 2:** The upper row gives the mean and standard deviation of all white matter microstructural indices for each of the three portions of the fornix in the Healthy ageing cohort. The lower row provides the Pearson r correlation coefficients between the tract-specific microstructural measures and age in the same cohort. Asterisks indicate significant correlations following Bonferroni adjustment (p ≤ 0.0014). Abbreviations: abFornix, anterior part of the body of the fornix; FA, fractional anisotropy; HA, hindrance modulated orientational anisotropy (HMOA); RD, radial diffusivity; MD, mean diffusivity; AD, axial diffusivity; f, tissue volume fraction. Index score = mean (standard deviation). Units for RD, MD and AD scores are given as × 10^3^ mm^2^ s^− 1^.

Healthy ageing cohort (n = 39)																		
	abFornix						Precommissural fornix						Postcommissural fornix					
HA	FA	RD	MD	AD	*f*	HA	FA	RD	MD	AD	*f*	HA	FA	RD	MD	AD	*f*
Mean	0.17	0.34	1.06	1.33	1.87	0.60	0.19	0.37	1.02	1.31	1.90	0.61	0.17	0.34	1.06	1.34	1.88	0.61
S.D.	0.03	0.02	0.11	0.13	0.18	0.04	0.04	0.03	0.10	0.12	0.17	0.05	0.04	0.04	0.12	0.14	0.21	0.04
Age (*r*)	− 0.25	− 0.13	0.60*	0.60*	0.58*	− 0.16	− 0.20	− 0.25	0.48	0.46	0.40	− 0.09	− 0.25	− 0.16	0.62*	0.60*	0.52*	− 0.31

**Table 3:** Pearson r correlations for all tract diffusion derived measures in Mild Cognitive Impairment and their Matched Control group. Asterisks indicate significant correlations following Bonferroni adjustment. The term abFornix refers to the anterior part of the body of the fornix. Abbreviations: FA, fractional anisotropy; HA, hindrance modulated orientational anisotropy (HMOA); RD, radial diffusivity; MD, mean diffusivity; AD, axial diffusivity; f, tissue volume fraction.

Mild Cognitive Impairment (n = 24)												
	Precommissural fornix						Postcommissural fornix					
abFornix	HA	FA	RD	MD	AD	f	HA	FA	RD	MD	AD	f
HA	0.47						0.31					
FA		0.74^**⁎**^						0.69^⁎^				
RD			0.86^⁎^						0.86^⁎^			
MD				0.75^⁎^						0.85^⁎^		
AD					0.55						0.76^⁎^	
*f*						0.63^⁎^						0.44

*Postcommissural fornix*												
HA	− 0.03											
FA		0.72^⁎^										
RD			0.80^⁎^									
MD				0.81^⁎^								
AD					0.60							
*f*						0.38						


Matched control group (n = 20)												
	Precommissural fornix						Postcommissural fornix					
abFornix	HA	FA	RD	MD	AD	f	HA	FA	RD	MD	AD	f
HA	0.58						0.72^⁎^					
FA		0.52						0.59^⁎^				
RD			0.92^⁎^						0.89^⁎^			
MD				0.94^⁎^						0.86^⁎^		
AD					0.91^⁎^						0.79^⁎^	
*f*						0.50						0.70^⁎^

*Postcommissural fornix*												
HA	0.21											
FA		0.22										
RD			0.75^⁎^									
MD				0.74^⁎^								
AD					0.65^⁎^							
*f*						0.01						

**Table 4:** Means and standard deviations for the anterior body of the fornix (abFornix), precommissural fornix, and postcommissural fornix for each diffusion derived index for the Mild Cognitive Impairment (MCI) and Matched Control groups. Abbreviations: FA, fractional anisotropy; HMOA, hindrance modulated orientational anisotropy; RD, radial diffusivity; f, tissue volume fraction.

	MCI (n = 24)	Matched control group (n = 20)
*abFornix*		
HMOA	0.14 (0.04)	0.17 (0.02)
FA	0.32 (0.04)	0.34 (0.02)
RD	1.06 (0.13) *x* *10*^*3*^	1.09 (0.12) *x* *10*^*3*^
MD	1.32 (0.15) *x* *10*^*3*^	1.37 (0.15) *x* *10*^*3*^
AD	1.84 (0.21) *x* *10*^*3*^	1.93 (0.21) *x* *10*^*3*^
*f*	0.58 (0.05)	0.59 (0.03)

*Precommissural fornix*		
HMOA	0.17 (0.04)	0.19 (0.04)
FA	0.33 (0.07)	0.36 (0.03)
RD	1.08 (0.12) *x* *10*^*3*^	1.05 (0.11) *x* 10^3^
MD	1.37 (0.13) *x* *10*^*3*^	1.34 (0.14) *x* *10*^*3*^
AD	1.95 (0.15) *x* *10*^*3*^	1.92 (0.21) *x* *10*^*3*^
*f*	0.59 (0.05)	0.61 (0.04)

*Postcommissural fornix*		
HMOA	0.18 (0.05)	0.17 (0.04)
FA	0.35 (0.07)	0.34 (0.04)
RD	1.08 (0.19) *x* *10*^*3*^	1.09 (0.14) *x* *10*^*3*^
MD	1.40 (0.21) *x* *10*^*3*^	1.38 (0.16) *x* *10*^*3*^
AD	2.03 (0.26) *x* *10*^*3*^	1.94 (0.22) *x* *10*^*3*^
*f*	0.57 (0.05)	0.60 (0.04)
